# Post-traumatic stress disorder in people with multiple sclerosis: A narrative review

**DOI:** 10.1177/13524585261475088

**Published:** 2026-08-05

**Authors:** David E Freedman, Jiwon Oh, Anthony Feinstein

**Affiliations:** Department of Psychiatry, Temerty Faculty of Medicine, University of Toronto, Toronto, ON, Canada; Department of Psychiatry, Sunnybrook Health Sciences Centre, Toronto, ON, Canada; Department of Psychiatry, St. Michael’s Hospital, Toronto, ON, Canada; Division of Neurology, Temerty Faculty of Medicine, University of Toronto, Toronto, ON, Canada; Division of Neurology, Department of Medicine, St. Michael’s Hospital, Toronto, ON, Canada; Department of Psychiatry, Temerty Faculty of Medicine, University of Toronto, Toronto, ON, Canada; Department of Psychiatry, Sunnybrook Health Sciences Centre, Toronto, ON, Canada; The BARLO MS Centre, St. Michael’s Hospital, Toronto, ON, Canada

**Keywords:** Multiple sclerosis, post-traumatic stress disorder, anxiety disorders, trauma

## Abstract

Psychiatric comorbidities are common in people with multiple sclerosis (pwMS). Yet, post-traumatic stress disorder (PTSD) has received little attention to date. Grounded in an emerging literature on the impact of trauma on pwMS, this Topical Review synthesizes current evidence on PTSD in pwMS. Although 5%–16% of pwMS have PTSD, there remains uncertainty about whether the prevalence exceeds that seen in the general population. Nonetheless, PTSD may influence measures of multiple sclerosis (MS)-related disease activity, employment, and participation in healthcare. Preliminary data indicate no sex difference in PTSD in pwMS, in contrast to an elevated risk in females in the general population. Further work is needed to clarify the demographic and disease-related correlates of PTSD, and the effectiveness of potential treatments in pwMS. By summarizing existing knowledge, this review highlights avenues for future research to advance care for people with comorbid PTSD and MS.

The impact of trauma and violence on the lives of people with multiple sclerosis (pwMS) is increasingly recognized. Adverse childhood experiences may be associated with an elevated risk of multiple sclerosis (MS) and other immune-mediated inflammatory diseases (compared to those unexposed to adverse childhood experiences),^1,2^ increased depressive symptoms,^3^ and an elevated risk of revictimization as adults.^1^ In adulthood, abuse is commonly experienced by women with MS,^4,5^ particularly those with advanced disease,^4^ highlighting the need for clinicians to appropriately address intimate partner violence in pwMS.^6^ Despite this, little is known about post-traumatic stress disorder (PTSD) in pwMS.

PTSD is defined by exposure to a traumatic event and subsequent development of trauma-associated intrusive symptoms, avoidance, negative alterations in cognition and mood, and symptoms of hyperarousal.^7^ Although PTSD may co-occur with anxiety disorders and depression, this condition is now recognized to have distinct trauma-related symptomatology, neurobiological correlates, and trauma-focused treatments.^8^ A brief note about what constitutes a “traumatic event” is relevant. The fifth edition of the *Diagnostic and Statistical Manual of Mental Disorders, Text Revision* (DSM-5-TR) characterizes a traumatic event as “exposure to actual or threatened death, serious injury, or sexual violence.”^7^ As explained in the DSM-5-TR text, “a life-threatening illness or debilitating medical condition is not necessarily considered a traumatic event,”^7^ and in this regard, “qualifying events of this type include life-threatening medical emergencies (e.g., an acute myocardial infarction, anaphylactic shock) or a particular event in treatment that evokes catastrophic feelings of terror, pain, helplessness, or imminent death (e.g. waking during surgery, debridement of severe burn wounds, emergency cardioversion).”^7^ Under this definition, a diagnosis with MS or routine treatment, although potentially stressful and even if perceived to be life-threatening, would not meet DSM-5-TR PTSD criteria for a traumatic event.

Psychiatric comorbidities are prevalent among pwMS.^9^ Despite expanding knowledge about the roles of trauma and violence in MS,^6^ no review has synthesized current knowledge on PTSD in pwMS. This Topical Review summarizes recent evidence about the prevalence, impact, demographic and disease-related correlates, and treatment of PTSD in pwMS, providing a primer for clinicians and a foundation for future research.

## Prevalence of PTSD in pwMS

In community-based samples, 5%–16% of pwMS have PTSD,^10–13^ with a higher prevalence (~17%) in veterans with MS.^14^ Heterogeneity in estimates of PTSD may be attributed to whether MS was determined from clinician diagnosis,^10–12^ self-report,^13^ or administrative health data,^14^ small samples,^10,12,13^ and non-consecutive sampling.^11–13^ Advantages of self-report scales include the low cost and ease of administration. These benefits may be particularly relevant in low-resource settings with limited availability of mental health professionals. However, the use of self-report scales may over-estimate the prevalence of PTSD,^15^ and self-report scales are not able to diagnose PTSD, emphasizing the value of clinical diagnoses or structured clinical interviews. Community-based administrative data frequently lump PTSD into a broad category of anxiety disorders,^9^ limiting the utility of these data sources. In addition, no study has estimated the prevalence of PTSD using structured interviews to assess the updated DSM-5-TR criteria. Although a diagnosis of MS accompanied by intense fear or helplessness would have qualified as a “traumatic event” in the *Diagnostic and Statistical Manual of Mental Disorders* (Fourth Edition; DSM-IV) diagnostic criteria for PTSD,^16^ under the *Diagnostic and Statistical Manual of Mental Disorders* (Fifth Edition; DSM-5) criteria, a diagnosis of MS no longer constitutes a “traumatic event.”^7^ This shift from DSM-IV, in part, has narrowed the definition of who meets DSM criteria for PTSD,^17^ curtailing the validity of current PTSD estimates in pwMS.

In civilian samples from the general population, the lifetime prevalence of PTSD is approximately 3% in males and 8% in females.^18^ These population estimates increase to 8% in males and 13% in females in veterans.^18^ Military-based administrative data indicate an elevated burden of MS among those with PTSD.^19,20^ A Swedish registry-based study further demonstrated a link between stress-related disorders (compared to matched individuals without stress-related disorders) and the subsequent development of multiple autoimmune diseases, including MS.^21^ Although a recent community-based investigation found no difference in PTSD symptom severity between pwMS and healthy controls, PTSD diagnoses were not evaluated, and data were gathered in the context of nearby conflicts.^22^ These issues complicate the interpretation of findings. Altogether, these data suggest a possible link between MS and PTSD, but there remains a need to rigorously evaluate this association in representative community-based samples using structured interviews with DSM-5-TR criteria.

## Potential mechanisms of a link between PTSD and MS

The association between pre-existing PTSD and the later occurrence of immune-mediated conditions was attributed to chronically low cortisol, heightened pro-inflammatory cytokines, accelerated immunological aging, or lifestyle changes.^21^ In alignment, psychiatric comorbidities may mediate the association between adverse childhood experiences and immune-mediated inflammatory diseases, including MS.^23^ However, this study did not specifically evaluate the mediating role of PTSD, an avenue for future research.

Shared risk factors may also explain an association between PTSD and MS. For example, a genome-wide association study of over one million participants found evidence in support of a weak correlation between genetic risk factors for PTSD and MS.^24^ Adverse childhood experiences may be the primary traumatic events in PTSD or linked to an increased risk of adult revictimization^1^ and are associated with an elevated risk of developing MS.^1,2^ It remains unknown whether MS influences one’s vulnerability to PTSD. Ascertaining the temporal order of onset between PTSD and MS will be crucial to begin clarifying the nature of a relationship.

## Impact of PTSD in pwMS

As expected, PTSD is linked to heightened symptoms of depression and anxiety in pwMS.^10,13,25^ In addition, PTSD is associated with an elevated risk of chronic opioid prescriptions,^26^ accessing pharmacotherapy support for smoking cessation,^14^ missed appointments,^27^ and unemployment in pwMS.^28^ Validation of these findings in independent samples remains necessary,^14,26–28^ but these data indicate connections between PTSD and recreational substance use or occupational difficulties in pwMS. Furthermore, in pwMS, pre-existing PTSD is linked to a heightened relapse rate, greater accumulation of new and gadolinium-enhancing lesions, and faster build-up of disability compared to those without a PTSD history.^29^ These data emphasize the potential marked connections between PTSD and both functional and radiological outcomes. Investigations are needed to clarify whether these are causal or associative relationships.

## Who is affected by PTSD?

As previously described, PTSD is more common in females than in males in the general population.^18^ Yet, in pwMS, preliminary data do not show an influence of sex/gender on rates of PTSD.^10,13,25^ These findings align with the absence of a difference in depressive symptoms between males and females with MS.^30^ An elevated burden of PTSD in women in the general population is often attributed to sex/gender differences in the risk of exposure to interpersonal violence (particularly in childhood), gender-specific influences of social environments, and a higher heritability of PTSD in females compared to males.^31^ It is unknown whether these factors differ in pwMS. Rigorous evaluation is needed to confirm whether the absence of a sex/gender difference in PTSD symptoms holds up under scrutiny.

In general, PTSD is more common in younger individuals and those with less education.^8^ Similar findings regarding age,^25^ and education,^10^ have been found in some studies of pwMS, but not all.^10,12,13^ Although disease duration does not influence PTSD symptoms,^10,12,13,25^ one study found a trend (not reaching significance) toward increased clinician-assessed physical disability and elevated odds of PTSD.^10^ There are mixed findings about whether PTSD is influenced by MS subtype.^12,13,25^ Larger investigations are needed to clarify what demographic or disease-related characteristics influence the risk of PTSD in pwMS.

## Treatment of PTSD

There are few studies to guide the treatment of PTSD in pwMS. In a pilot randomized controlled trial of eye movement desensitization and reprocessing (EMDR) in 42 pwMS, a greater proportion of participants who received EMDR compared to relaxation therapy did not meet criteria for PTSD 6 months after treatment, considered to be a marginally statistically significant difference on the primary outcome measure.^32^ However, there was no statistically significant interaction between pre–post symptom differences and assigned treatment arm.^32^ This study is further limited by its small sample.^32^ Guidelines from the general population also support the use of trauma-focused therapies, such as EMDR, cognitive processing therapy, or prolonged exposure therapy, to address PTSD.^33^ There are no other studies of trauma-focused therapies in people with comorbid PTSD and MS.

A case report describes visual hallucinations in an older adult with PTSD and MS who was treated with bupropion for fatigue.^34^ The complexity of this individual case description makes it challenging to draw a definitive conclusion about pharmacotherapy in people with comorbid PTSD and MS. There are no observational or experimental studies of pharmacotherapy for PTSD in pwMS. This is a notable gap considering the potential efficacy of medications, such as paroxetine, sertraline, venlafaxine, and prazosin, to manage PTSD symptoms in the general population^33^ and limited access to evidence-based psychotherapy in many clinical settings.^35^ A prior investigation suggested that use of selective serotonin reuptake inhibitors may be linked to a diminished association between stress-related disorders and autoimmune disease.^21^ Furthermore, fatigue, balance difficulties, and cognitive dysfunction are common symptoms of MS and possible side effects of these psychotropic medications. These findings emphasize the need to evaluate the efficacy and tolerability of these medications in pwMS who have PTSD, along with the impact on disease activity.

## Limitations

There are a few caveats to consider in interpreting this review. A narrative approach was selected in light of limited research to date and to enable an in-depth exploration of the evidence base with context from studies in the general population. In addition, only English articles were included. Finally, there are few studies on people with comorbid PTSD and MS, emphasizing the need for this review as a framework for future work.

## Conclusion

Mindful of these limitations, this review suggests that PTSD is common in pwMS and may adversely influence work, engagement with healthcare, and MS-related radiological outcomes. Many questions, however, remain unanswered. These include whether there is a consistent association between PTSD and MS, whether there are neuroimaging or neurocognitive correlates of PTSD symptoms in pwMS, how sex- and gender-related factors influence PTSD risk, and whether certain demographic or disease-related characteristics are linked to greater vulnerability to PTSD in pwMS. Furthermore, pharmacotherapy or psychotherapy trials may clarify whether the successful treatment of PTSD in pwMS averts previously noted negative outcomes. Despite these areas of uncertainty, this review sheds light on what is known about this debilitating condition and paves a pathway forward to improve the recognition and management of PTSD in pwMS.

## Data Availability

Data sharing is not applicable to this article as no datasets were generated or analyzed during the current study.

## References

[1] Eid K Torkildsen Ø Aarseth J , et al. Association of adverse childhood experiences with the development of multiple sclerosis. J Neurol Neurosurg Psychiatry 2022; 93(6): 645–650. 10.1136/jnnp-2021-328700 PMC9148981 35379699

[2] O’Mahony J Bernstein CN Marrie RA. A nested case-control study evaluating the relationship between adverse childhood experiences and immune-mediated inflammatory disease in the Canadian longitudinal study on aging. BMJ Open 2025; 15(1): e087133. 10.1136/bmjopen-2024-087133 PMC11749297 39833007

[3] Guerrero KS Horton MK Choudhary V , et al. Adverse childhood experiences in early life increase the odds of depression among adults with multiple sclerosis. Mult Scler J Exp Transl Clin 2023; 9(4): 20552173231202638. 10.1177/20552173231202638 PMC10552460 37808459

[4] Pol-Patil J Glanz B Safar L , et al. MeTooMS: Sexual, physical, and emotional abuse experience among women with multiple sclerosis. Mult Scler 2023; 29(2): 287–294. 10.1177/13524585221122169 36154526

[5] Etemadifar M Zamani O Bahrami-Zadegan A , et al. Intimate partner violence among women with multiple sclerosis. PCN Rep 2025; 4(4): e70242. 10.1002/pcn5.70242 PMC12605950 41234222

[6] Freedman DE Krysko KM Feinstein A. Intimate partner violence and multiple sclerosis. Mult Scler 2024; 30(3): 295–298. 10.1177/13524585231197926 PMC10935614 37732567

[7] American Psychiatric Association. Diagnostic and statistical manual of mental disorders. 5th ed. Washington, DC: American Psychiatric Association Publishing, 2022.

[8] Shalev A Cho D Marmar CR. Neurobiology and treatment of posttraumatic stress disorder. Am J Psychiatry 2024; 181(8): 705–719. 10.1176/appi.ajp.20240536 39086292

[9] Marrie RA Reingold S Cohen J , et al. The incidence and prevalence of psychiatric disorders in multiple sclerosis: A systematic review. Mult Scler 2015; 21(3): 305–317. 10.1177/1352458514564487 PMC4429164 25583845

[10] Ostacoli L Carletto S Borghi M , et al. Prevalence and significant determinants of post-traumatic stress disorder in a large sample of patients with multiple sclerosis. J Clin Psychol Med Settings 2013; 20(2): 240–246. 10.1007/s10880-012-9323-2 23053829

[11] Carletto S Borghi M Scavelli F , et al. Prevalence of posttraumatic stress disorder in patients with multiple sclerosis. J Nerv Ment Dis 2018; 206(2): 149–151. 10.1097/NMD.0000000000000780 29373457

[12] Chalfant AM Bryant RA Fulcher G. Posttraumatic stress disorder following diagnosis of multiple sclerosis. J Trauma Stress 2004; 17(5): 423–428. 10.1023/B:JOTS.0000048955.65891.4c 15633921

[13] Heidler F Hecker M Frahm N , et al. Trauma burden affected people with multiple sclerosis during SARS-CoV-2 pandemic. J Clin Med 2025; 14(8): 2665. 10.3390/jcm14082665 PMC12027752 40283495

[14] Polick CS Dennis P Calhoun PS , et al. Investigating disparities in smoking cessation treatment for veterans with multiple sclerosis: A national analysis. Brain Behav 2024; 14(5): e3513. 10.1002/brb3.3513 PMC11066415 38698620

[15] Schincariol A Orrù G Otgaar H , et al. Posttraumatic stress disorder (PTSD) prevalence: An umbrella review. Psychol Med 2024; 54(15): 4021–4034. 10.1017/S0033291724002319 PMC11650175 39324396

[16] American Psychiatric Association. Diagnostic and statistical manual of mental disorders. 4th ed. Washington, DC: American Psychiatric Association Publishing, 1994.

[17] Kilpatrick DG Resnick HS Milanak ME , et al. National estimates of exposure to traumatic events and PTSD prevalence using DSM-IV and DSM-5 criteria. J Trauma Stress 2013; 26(5): 537–547. 10.1002/jts.21848 PMC4096796 24151000

[18] Schein J Houle C Urganus A , et al. Prevalence of post-traumatic stress disorder in the United States: A systematic literature review. Curr Med Res Opin 2021; 37(12): 2151–2161. 10.1080/03007995.2021.1978417 34498953

[19] O’Donovan A Cohen BE Seal KH , et al. Elevated risk for autoimmune disorders in Iraq and Afghanistan veterans with posttraumatic stress disorder. Biol Psychiatry 2015; 77(4): 365–374. 10.1016/j.biopsych.2014.06.015 PMC4277929 25104173

[20] Bookwalter DB Roenfeldt KA LeardMann CA , et al. Posttraumatic stress disorder and risk of selected autoimmune diseases among US military personnel. BMC Psychiatry 2020; 20(1): 23. 10.1186/s12888-020-2432-9 PMC6964079 31941473

[21] Song H Fang F Tomasson G , et al. Association of stress-related disorders with subsequent autoimmune disease. JAMA 2018; 319(23): 2388–2400. 10.1001/jama.2018.7028 PMC6583688 29922828

[22] Al-Ameri M Mansour M Al-Habahbeh S , et al. Symptoms of PTSD and depression in newly diagnosed people with multiple sclerosis during the outbreak Oct. Mult Scler Relat Disord 2025; 93: 106206. 10.1016/j.msard.2024.106206 39631135

[23] O’Mahony J Bernstein CN Marrie RA. Adverse childhood experiences and psychiatric comorbidity in multiple sclerosis, inflammatory bowel disease, and rheumatoid arthritis in the Canadian longitudinal study on aging. J Psychosom Res 2024; 187: 111893. 10.1016/j.jpsychores.2024.111893 39306899

[24] Li M Qu K Wang Y , et al. Associations between post-traumatic stress disorder and neurological disorders: A genetic correlation and Mendelian randomization study. J Affect Disord 2025; 370: 547–556. 10.1016/j.jad.2024.11.044 39547276

[25] Counsell A Hadjistavropoulos HD Kehler MD , et al. Posttraumatic stress disorder symptoms in individuals with multiple sclerosis. Psychol Trauma Theor Res Pract Policy 2013; 5(5): 448.

[26] Turner AP Arewasikporn A Hawkins EJ , et al. Risk factors for chronic prescription opioid use in multiple sclerosis. Arch Phys Med Rehabil 2023; 104(11): 1850–1856. 10.1016/j.apmr.2023.04.012 37137460

[27] Gromisch ES Turner AP Leipertz SL , et al. Who is not coming to clinic? A predictive model of excessive missed appointments in persons with multiple sclerosis. Mult Scler Relat Disord 2020; 38: 101513. 10.1016/j.msard.2019.101513 31756611

[28] Gammoh O Qnais E Abu Alshaikh H , et al. PTSD symptoms among patients with multiple sclerosis in Jordan during October 7th Gaza war outbreak. Int J Psychiatry Med 2025; 60(1): 7–16. 10.1177/00912174241272680 39106247

[29] Leekoff M Culpepper W Jin S , et al. Impact of comorbid post traumatic stress disorder on multiple sclerosis in military veterans: A population-based cohort study. Mult Scler 2022; 28(8): 1257–1266. 10.1177/13524585211058361 34854320

[30] Freedman DE Oh J Einstein G , et al. Sex differences in the neuropsychiatric symptoms of multiple sclerosis: Symptômes neuropsychiatriques de la sclérose en plaques: Différences selon le sexe. Can J Psychiatry 2026; 71(8): 612–622. 10.1177/07067437261425067 PMC12949738 41762167

[31] Kimerling R Allen MC Duncan LE. Chromosomes to social contexts: Sex and gender differences in PTSD. Curr Psychiatry Rep 2018; 20(12): 114. 10.1007/s11920-018-0981-0 30345456

[32] Carletto S Borghi M Bertino G , et al. Treating post-traumatic stress disorder in patients with multiple sclerosis: A randomized controlled trial comparing the efficacy of eye movement desensitization and reprocessing and relaxation therapy. Front Psychol 2016; 7: 526. 10.3389/fpsyg.2016.00526 PMC4838623 27148134

[33] U.S. Department of Veterans Affairs. VA/DoD clinical practice guideline for management of posttraumatic stress disorder and acute stress disorder. U.S. Government Printing Office, 2023.

[34] Kojima G Tamai A Karino S , et al. Bupropion-related visual hallucinations in a veteran with posttraumatic stress disorder and multiple sclerosis. J Clin Psychopharmacol 2013; 33(5): 717–719. 10.1097/JCP.0b013e3182a11622 23963062

[35] Freedman DE Oh J Feinstein A. Not for everyone: Factors influencing who receives psychotherapy in people with multiple sclerosis. Mult Scler Relat Disord 2025; 94: 106245. 10.1016/j.msard.2024.106245 39764908

